# Cascade source inference in networks: a Markov chain Monte Carlo approach

**DOI:** 10.1186/s40649-015-0017-4

**Published:** 2015-10-19

**Authors:** Xuming Zhai, Weili Wu, Wen Xu

**Affiliations:** 1 College of Computer Science and Technology, Taiyuan University of Technology, Taiyuan, 030024 China; 2 Department of Computer Science, University of Texas at Dallas, 800 W. Campbell Rd, Richardson, 75080 TX USA

**Keywords:** Social network, Source inference, Markov chain Monte Carlo

## Abstract

Cascades of information, ideas, rumors, and viruses spread through networks. Sometimes, it is desirable to find the source of a cascade given a snapshot of it. In this paper, source inference problem is tackled under Independent Cascade (IC) model. First, the #P-completeness of source inference problem is proven. Then, a Markov chain Monte Carlo algorithm is proposed to find a solution. It is worth noting that our algorithm is designed to handle large networks. In addition, the algorithm does not rely on prior knowledge of when the cascade started. Finally, experiments on real social network are conducted to evaluate the performance. Under all experimental settings, our algorithm identified the true source with high probability.

## Introduction

Modern social and computer networks are common media for cascades of information, ideas, rumors, and viruses. It is often desirable to identify the source of a cascade from a snapshot of the cascade. For example, a good way to stop a rumor is to find out the person that has fabricated it. Similarly, identifying the first computer infected by a virus provides valuable information for catching the author. Therefore, given the network structure and an observed cascade snapshot consisting only the set of infected/active nodes, solving the source inference problem is very useful in many cases. Hereafter, we use infected/active and infect/activate interchangeably.

In the seminal works [1] and [2], source inference problem under susceptible-infected (SI) model is first studied, and a maximum likelihood estimator is proposed with theoretical performance bound when the network is a tree. Based on the same model, many works solve this problem with different extensions. With a priori knowledge of a candidate source set, reference [3] infers the source node using a maximum a posteriori estimator. Wang et al. [4] utilizes multiple independent epidemic observations to single out their common source. Karamchandani and Franceschetti [5] study the case where infected nodes reveal their infection with a probability. When multiple sources are involved, algorithms are proposed in [6] and [7] to find out all of them. The works mentioned above, except [7], are all based on tree networks, while some of them are applicable to general graphs by constructing breadth-first-search trees. More importantly, all of them use SI model, where an infected node will certainly infect a susceptible neighbor after a random period of time. Our work, however, is based on Independent Cascade (IC) model. In the IC model, an active node activates its successor with a certain probability determined by the edge weight.

Although SI model is popular in epidemiological researches because it catches the pattern of epidemics, the IC model is arguably more suitable to depict cascades in social networks, where relationship between peers plays a more important role than time of infection. As an example, suppose Alice bought a new hat, her classmates may or may not imitate the purchase depending on how they agree with her taste. Those who do not appreciate her taste are unlikely to change their minds even Alice wears her hat every day. These people are now immune from the influence of Alice’s new hat, though they may still be persuaded by someone they appreciate more.

**Fig. 1:**
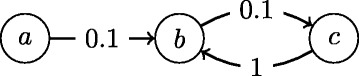
Example of a simple case of source inference problem: if all three nodes are found active, then node *a* must be the source

Although the IC model is popular in social network researches, finding source in the IC model is rarely studied. Using a model similar to the IC with identical edge weight, reference[8] studies the problem of inferring both links and sources given multiple observed cascades. Under the IC model, reference [9] solves the problem of finding sources that are expected to generate cascades most similar to the observation. Surprisingly, this problem is fundamentally different from source inference problem, which finds the source that most likely has started the observed cascade. For example, when a cascade that infects all nodes is observed in the simple linear network in Fig. 1, node *c* is the optimal result for the problem defined in [9] because it is expected to generate a cascade with least difference from the observed one. However, it is obvious that *c* cannot be responsible for a cascade that spreads through all three nodes.

In this paper, we work on the problem of detecting the source node that is responsible for a given cascade. We first formulate the source inference problem in the IC model and prove its #P-completeness. Then, a Markov chain Monte Carlo (MCMC) algorithm is proposed to solve the inference problem. It is worth noting that our algorithm scales with observed cascade size rather than network size, which is very important due to the huge size of social networks nowadays. Another advantage of our algorithm is that it is designed to deal with snapshot of cascades taken either before or after termination. More importantly, our algorithm does not require prior knowledge of the starting time of the cascade, which is usually unknown in practical scenarios. To evaluate the performance of our algorithm, experiments are done in a real network. Experimental results demonstrate the effectiveness of our algorithm.

## Problem formulation

### Propagation model

In this work, we model a social network as a weighted directed graph *G*(*V*,*E*) with weights *w*_*i*,*j*_∈(0,1] associated with each edge (*i*,*j*)∈*E* representing the probability of *i* successfully influencing *j*. The propagation procedure of a cascade in the network is depicted by the well-known IC model [10]. The cascade starts with all nodes inactive except a source node *s*, which we assume is activated at time *τ*_0_. At every time step *τ*>*τ*_0_, every node *i* that was activated at *τ*−1 has a single chance to influence each of its inactive successors through the directed edge with success probability specified by the weight of the edge. If the influence is successful, then the successor is activated at time *τ* and will be able to influence its inactive successors at the next time step. The process terminates when no new node is activated.

An important fact about the IC model is that each active node has only one chance of influencing each of its neighbors. To put it another way, there is only one chance for each edge to participate in the propagation with success rate specified by the weight. Since edge weights are fixed and independent of the cascade, we can flip the biased coins even before the cascade starts to determine whether each edge will help the propagation. This gives an alternative process consisting of two steps that also simulates the IC model. First, a subgraph *G* ^′^ of the original network *G* is taken by 1) keeping all vertices and 2) filtering edges according to their weights, i.e.,

(1) $$ \begin{aligned} \forall v\in G,&\quad v\in G^{\prime},\\ \forall (i,j)\in G,&\quad \text{Pr}\,{((i,j) \in G^{\prime})} = w_{i,j}. \end{aligned}   $$

Then, every node *i* reachable from source *s* in *G* ^′^ is active, with its activation time set to $\tau + d_{G^{\prime }}(s,i)$, where $d_{G^{\prime }}(s,i)$ is the distance, i.e., number of edges in the directed shortest path, from *s* to *i* in *G* ^′^.

It is easy to verify that the alternative process is equivalent to the previous one. Moreover, the alternative view builds the equivalence between sampling subgraphs of network and simulating cascades on it. Due to this convenience, we extensively use the alternative view in the following sections.

### Source inference problem

Suppose in a given network *G*, an unnoticed cascade starts from an unknown source node *s* ^∗^ at time *τ*_0_. Later at time *τ*_0_+*τ*, the cascade is discovered and the set of active nodes *A*_*τ*_ is identified without knowing their corresponding activation time. Note that *A*_*τ*_ can be viewed as a snapshot of the cascade at time *τ*. Now, we want to find the node $\hat s$ that most likely had started the cascade. Thus,

(2) $$ \hat s = \arg\max_{s} \text{Pr}\,({A_{\tau}}|{G,s,\tau}) ,  $$

where Pr (*A*_*τ*_|*G*,*s*,*τ*) denotes the probability of a cascade on *G* starting from *s* having snapshot *A*_*τ*_ at time *τ*. According to the alternative view of the IC model defined in the ‘Propagation model’ section and suppose *G* ^′^ is sampled according to (1), we have

(3) $$ \text{Pr}\,({A_{\tau}}|{G,s,\tau}) = \text{Pr}\,({A_{\tau}=\{i\mid d_{G^{\prime}}(s,i)\le\tau\}}).  $$

The following theorem shows the intractability of source inference problem, i.e., solving (2) given *G*, *τ*, and *A*_*τ*_.

#### **Theorem1**.

Source inference problem is #P-complete.

This theorem is proven by constructing a polynomial-time Turing reduction from s-t connectedness problem [11] to source inference problem. Please refer to Appendix 1 for the detailed proof.

## Source inference algorithm

### Basic algorithm

We use $\mathcal {R}(G^{\prime },s,\tau)$ to denote the set of nodes in *G* ^′^ reachable from *s* within distance *τ*, i.e.,

$$\mathcal{R}(G^{\prime},s,\tau) = \{i\mid d_{G^{\prime}}(s,i)\le\tau\}.  $$

Then, the probability shown in (3) can also be written as

(4) $$\begin{array}{*{20}l} {\text{Pr}}\,({A_{\tau}}|{G,s,\tau}) &= \sum_{G^{\prime}\subseteq G} {\text{Pr}}_{\mathcal{G}}({G^{\prime}}) I(A_{\tau} = {\mathcal{R}}(G^{\prime},s,\tau))  \end{array} $$

(5) $$\begin{array}{*{20}l} &= {\mathbb{E}}_{{G^{\prime}\sim\mathcal{G}}}[{I(A_{\tau} = {\mathcal{R}}(G^{\prime},s,\tau))}],  \end{array} $$

where  represents the distribution of subgraphs of *G* defined by (1), ${\text {Pr}}_{\mathcal {G}}({G^{\prime }})$ denotes the probability mass function (PMF) of *G* ^′^ in distribution , i.e.,

(6) $$ {\text{Pr}}_{\mathcal{G}}({G^{\prime}}) = \prod_{(i,j)\in G}w_{i,j}^{I((i,j)\in G^{\prime})}(1-w_{i,j})^{I((i,j)\notin G^{\prime})}   $$

and *I* is an indicator function defined as

$$I(c) = \left\{\begin{array}{ll} 1 & \text{if condition} ~~c~~ \text{is true},\\ 0 & \text{otherwise}. \end{array}\right.  $$

Because of the #P-completeness of source inference problem, calculating exact value of (4) is #P-hard.

A trivial method to approximate the value is to estimate the expectation in (2) by randomly sampling graphs in . But this method is still impractical. To show this, we define $\mathcal {S} = \{G^{\prime }\mid G^{\prime }\subseteq G\}$ as the set of all subgraphs of *G*, which is also the support of . Then, a subset of  is defined as

$$\mathcal{S}^{\prime} = \{G^{\prime}\mid G^{\prime}\subseteq G, \exists s, s\leadsto A_{\tau} \subseteq G^{\prime}\}, $$

where *s*⇝*A*_*τ*_⊆*G* ^′^ denotes “every node in *A*_*τ*_ is reachable from *s* in *G* ^′^”. Now, notice that $A_{\tau } = \mathcal {R}(G^{\prime },s,\tau) \Longrightarrow G^{\prime } \in \mathcal {S}^{\prime }$ and that the ratio $|\mathcal {S}|/|\mathcal {S}^{\prime }|$ can be exponential to |*G*|, which means almost all subgraphs of *G* will make the indicator function in equals 0. As an example, consider a linear graph *G*_*L*_(*V*_*L*_,*E*_*L*_) where *V*_*L*_={*v*_1_,*v*_2_,…,*v*_*n*_} and *E*_*L*_={(*v*_*k*_,*v*_*k*+1_)∣1≤*k*<*n*}. Suppose *A*_*τ*_=*V*_*L*_ and *τ*=*n*, then $|\mathcal {S}|=2^{n-1}$ whereas *s*⇝*A*_*τ*_⊆*G* ^′^ only if $G^{\prime }_{L} = G_{L}$ and *s*=*v*_1_.

To overcome this problem, we want to sample *G* ^′^ from set $\mathcal {S}^{\prime }$ rather than . On set $\mathcal {S}^{\prime }$, we define a new sampling distribution, denoted as $\mathcal {G}^{\prime }$, whose PMF is

(7) $$ \begin{aligned} \text{Pr}_{\mathcal{G}^{\prime}}({G^{\prime}}) &= \left\{\begin{array}{ll} \text{Pr}_{\mathcal{G}^{\prime}}({G^{\prime}}) / Z & {if } G^{\prime}\in \mathcal{S}^{\prime},\\ 0 & \text{otherwise}, \end{array}\right.\\ Z &= \sum_{G^{\prime}\in \mathcal{S}^{\prime}} \text{Pr}_{\mathcal{G}^{\prime}}({G^{\prime}}). \end{aligned}  $$

Notice that set $\mathcal {S}^{\prime }$ is independent of any candidate source node, so is the normalization factor *Z*. Therefore, with (7), we have

$$\mathbb{E}_{G^{\prime}\sim\mathcal{G}}[I(A_{\tau} = \mathcal{R}(G^{\prime},s,\tau))] \propto \mathbb{E}_{G^{\prime}\sim\mathcal{G}^{\prime}}[I(A_{\tau} = \mathcal{R}(G^{\prime},s,\tau))]. $$

Consequently, we can solve source inference problem (2) by solving

(8) $$ \hat s = \arg\max_{s} \mathbb{E}_{G^{\prime}\sim\mathcal{G}^{\prime}}[I(A_{\tau} = \mathcal{R}(G^{\prime},s,\tau))].  $$

Now the problem is how to sample from $\mathcal {S}^{\prime }$ with probability defined in (7). However, one can easily show that calculating factor *Z* is #P-hard, which makes calculating (7) impractical. Therefore, it is unlikely to be possible to directly sample from set $\mathcal {S}^{\prime }$. Fortunately, the probability ratio between any two subgraphs is easy to compute; thus, we can use Metropolis algorithm to sample distribution $\mathcal {G}^{\prime }$ in a Markov chain Monte Carlo.



Algorithm 1 describes a local move from a subgraph in $\mathcal {S}^{\prime }$ to another. Each local move will add/remove an edge to/from the previous subgraph $G^{\prime }_{k}$. The new subgraph $G^{\prime }_{k+1}$ is either accepted or rejected depending on the probability ratio $\text {Pr}_{\mathcal {G}^{\prime }}(G^{\prime }_{k+1})/ \text {Pr}_{\mathcal {G}^{\prime }}({G^{\prime }_{k}})$ defined in $\mathcal {G}^{\prime }$. Starting from any subgraph in $\mathcal {S}^{\prime }$, running Algorithm 1 iteratively will produce a Markov chain whose states represent subgraphs in $\mathcal {S}^{\prime }$ and whose stationary distribution is exactly the same as (7).

With the help of local move in Algorithm 1, Algorithm 2 infers the most likely source node responsible for the cascade snapshot *A*_*τ*_ taken at time *τ*. Input parameter *K* is used to indicate the number of samples to take by this algorithm. With line ??, the algorithm starts with whole graph *G* as the initial sample, which is obviously in $\mathcal {S}^{\prime }$. During every iteration of the while-loop, a subgraph in $\mathcal {S}^{\prime }$ is sampled, and all possible source vertices are found and recorded. After the while-loop ends, *c* *o* *u* *n* *t*[*i*]/*K* is the estimation of $\mathbb {E}_{G^{\prime }\sim \mathcal {G}^{\prime }}[{I(A_{\tau } = \mathcal {R}(G^{\prime },i,\tau))}]$. Hence, the returned value of Algorithm 2 is an approximate solution of (8).



### A more practical approach

Algorithm 2 has some drawbacks in practical scenarios. First, the whole network may be orders of magnitude larger than the cascade snapshot in question. However, Algorithm 2 scales with the size of full network rather than the snapshot, which is unfavorable here. Second, when the source node of a cascade is unknown, the starting time of the cascade is usually also absent. In these cases, inferring source node without knowing *τ* is desired. In this section, we will handle these two problems.

Based on the cascade snapshot *A*_*τ*_, we can classify edges in *E* into three disjoint subsets

(9) $$\begin{array}{*{20}l} E_{1} &= \{(i,j)\mid (i,j)\in E, i,j \in A_{\tau}\},\\ E_{2} &= \{(i,j)\mid (i,j)\in E, i \in A_{\tau}, j\notin A_{\tau}\},\\ E_{3} &= \{(i,j)\mid (i,j)\in E, i \notin A_{\tau}\}. \end{array} $$

And *E*_2_ can be further split into subsets according to the source node of edges:

$$ E_{2,u} = \{(i,j)\mid (i,j)\in E_{2}, i=u\}.  $$

Then we define three subgraphs of *G*(*V*,*E*) accordingly: *G*_1_(*A*_*τ*_,*E*_1_), *G*_2_(*V*,*E*_2_), and *G*_3_(*V*,*E*_3_). Note that *G*_1_ only contains nodes in *A*_*τ*_ because edges in *G*_1_ are all between nodes in *A*_*τ*_. Furthermore, we partition each sampled subgraph *G* ^′^ into $G^{\prime }_{1}$, $G^{\prime }_{2}$ and $G^{\prime }_{3}$, where $G^{\prime }_{k} = G^{\prime }\cap G_{k}$. With these definitions, we have the following lemma.

#### **Lemma1**.

If we define subgraph *G*_1_(*A*_*τ*_,*E*_1_)consisting of only edges between nodes in *A*_*τ*_, the condition

(10) $$ A_{\tau}=\mathcal{R}(G^{\prime},s,\tau)   $$

is equivalent to the combination of

(11) $$ A_{\tau}=\mathcal{R}(G^{\prime}_{1},s,\tau)   $$

and

(12) $$ \forall i\in A_{\tau},\quad d_{G^{\prime}_{1}}(s,i) = \tau \;\vee\; E_{2,i} \cap E^{\prime} = \varnothing,   $$

where $G^{\prime }_{1} = G^{\prime } \cap G_{1}$.

#### *Proof*.

Eq. 10 can be split to 1) any node in *A*_*τ*_ must be within distance *τ* from *s*, i.e.,

(13) $$ A_{\tau} \subseteq \mathcal{R}(G^{\prime},s,\tau),   $$

and 2) any node outside *A*_*τ*_ must have distance from *s* larger than *τ*, i.e.,

(14) $$ \mathcal{R}(G^{\prime},s,\tau)\setminus A_{\tau} = \varnothing.   $$

Hence, the shortest path from *s* to any node *i*∈*A*_*τ*_ is within *G*_1_, which implies ∀*i*∈*A*_*τ*_, $d_{G^{\prime }}(s,i) = d_{G^{\prime }_{1}}(s,i)$ and thus (11). Further, (12) means any node *i* with $d_{G^{\prime }}(s,i) < \tau $ must not be able to activate its neighbors outside *A*_*τ*_, which is necessary to ensure (14).

On the other hand, (11) guarantees (13) and (12) ensures $\forall i\notin A_{\tau }, d_{G^{\prime }}(s,i)>\tau $ which leads to (14).

From Lemma 1, it is straightforward to get the following corollaries.

#### **Corollary1**.

The indicator function in (4) is equivalent to

$$\begin{aligned} I(A_{\tau}=\mathcal{R}(G^{\prime},s,\tau)) =& I(A_{\tau}=\mathcal{R}(G^{\prime}_{1},s,\tau))\\ &\cdot \prod_{(i,j)\in G^{\prime}_{2}} I(d_{G^{\prime}_{1}}(s,i) = \tau). \end{aligned} $$

#### **Corollary2**.

$I(A_{\tau }=\mathcal {R}(G^{\prime },s,\tau))$ is independent of $G^{\prime }_{3}$.

In addition, because $G^{\prime } = G^{\prime }_{1}\cup G^{\prime }_{2}\cup G^{\prime }_{3}$ and edge sets in $G^{\prime }_{k}$ are disjoint, (6) can be rewritten as the product of three terms

(15) $$ \begin{aligned} \text{Pr}_{\mathcal{G}}({G^{\prime}}) &= \prod_{(i,j)\in G}w_{i,j}^{I((i,j)\in G^{\prime})}(1-w_{i,j})^{I((i,j)\notin G^{\prime})}\\ &= \prod_{k=1}^{3}\text{Pr}{_{\mathcal{G}}{_{k}}}({G^{\prime}_{k}}), \end{aligned}   $$

where

(16) $$ \text{Pr}_{{\mathcal{G}}_{k}}({G^{\prime}_{k}}) = \prod_{(i,j)\in G_{k}}w_{i,j}^{I((i,j)\in G^{\prime}_{k})}(1-w_{i,j})^{I((i,j)\notin G^{\prime}_{k})}.   $$

Now we have Theorem 2 that speedup the algorithm.

#### **Theorem2**.

Define distribution $\mathcal {G}^{\prime }_{1}$ of graphs in $\mathcal {S}^{\prime }_{1} = \{G^{\prime }_{1} \mid G^{\prime }_{1}\subseteq G_{1}, \exists s, s\leadsto A_{\tau } \subseteq G^{\prime }_{1}\}$ with PMF proportional to $\text {Pr}_{\mathcal {G}_{1}}({G^{\prime }_{1}})$. Then, we have

(17) $$ \text{Pr}\,(A_{\tau}|{G,s,\tau}) \propto \mathbb E_{G^{\prime}_{1}\sim \mathcal{G}^{\prime}_{1}} [f(G^{\prime}_{1},s,\tau)],  $$

where

(18) $$ f(G^{\prime}_{1},s,\tau) =I(A_{\tau} = \mathcal{R}(G^{\prime}_{1},s,\tau)) \prod_{\substack{(i,j)\in G_{2}\\ d_{G^{\prime}_{1}}(s,i) < \tau}}(1-w_{i,j}).   $$

The proof of Theorem 2 is shown in Appendix 2.

Theorem 2 shows that sampling subgraphs of *G*_1_, rather than the whole network *G*, is sufficient to infer the cascade source, which greatly accelerates the algorithm when the whole network is much larger than the cascade snapshot *A*_*τ*_.

Next, we deal with unknown cascade starting time, i.e., unknown *τ*. First, due to the fact that node set in *G*_1_ is *A*_*τ*_,

$$\begin{aligned} A_{\tau} = \mathcal{R}(G^{\prime}_{1},s,\tau) &\Longleftrightarrow A_{\tau} \subseteq \mathcal{R}(G^{\prime}_{1},s,\tau)\\ &\Longleftrightarrow s \leadsto A_{\tau} \subseteq G^{\prime}_{1} \wedge \tau \ge \epsilon_{G^{\prime}_{1}}(s), \end{aligned} $$

where $\epsilon _{G^{\prime }_{1}}(s)$ is the eccentricity of node *s* in $G^{\prime }_{1}$, defined as

(19) $$ \epsilon_{G^{\prime}_{1}}(s)=\max_{i\in G^{\prime}_{1}}d_{G^{\prime}_{1}}(s,i).  $$

As a result, for any given $G^{\prime }_{1}$ and *s* such that $s \leadsto A_{\tau } \subseteq G^{\prime }_{1}$, there are three possible values for function *f*(*G* ^′^,*s*,*τ*) in (18):

(20) $$ f(G^{\prime},s,\tau) = \left\{\begin{array}{ll} 0, &\tau < \epsilon_{G^{\prime}_{1}}(s),\\ \prod\limits_{\substack{(i,j)\in G_{2}\\d_{G^{\prime}_{1}}(s,i) < \epsilon_{G^{\prime}_{1}}(s)}} (1-w_{i,j}), &\tau = \epsilon_{G^{\prime}_{1}}(s),\\ \prod\limits_{\substack{(i,j)\in G_{2}}}(1-w_{i,j}), & \tau > \epsilon_{G^{\prime}_{1}}(s). \end{array}\right.   $$

Here, the values for all three cases are independent of *τ*. Then, we have Theorem 3 that deals with unknown cascade starting time.

#### **Theorem3**.

Suppose samples $G^{\prime }_{1,k}$, *k*=1,2,…,*K* are taken with distribution $\mathcal {G}^{\prime }_{1}$, then we can approximate (17) by

$$\mathbb E_{G^{\prime}_{1}\sim\mathcal{G}^{\prime}_{1}}[f(G^{\prime}_{1},s,\tau)] \approx \frac{1}{K} \left(A(s,\tau) + W\cdot\sum_{\tau^{\prime}<\tau}C(s,\tau^{\prime})\right), $$

where

(21) $$\begin{array}{*{20}l} A(s,\tau) &= \sum_{k:\tau=\epsilon_{G^{\prime}_{1,k}}(s)} \prod_{\substack{(i,j)\in G_{2}\\ d_{G^{\prime}_{1,k}}(s,i) < \epsilon_{G^{\prime}_{1,k}}(s)}}(1-w_{i,j}),\\ W &= \prod_{\substack{(i,j)\in G_{2}}}(1-w_{i,j}),\\ C(s,\tau^{\prime}) &= \sum_{k:\tau^{\prime}=\epsilon_{G^{\prime}_{1,k}}(s)} 1. \end{array} $$

#### *Proof*.

Because samples $G^{\prime }_{1,k}$ are taken with distribution $\mathcal {G}^{\prime }_{1}$, we have

(22) $$ \mathbb E_{G^{\prime}_{1}\sim\mathcal{G}^{\prime}_{1}}[\;\!f(G^{\prime}_{1},s,\tau)] \approx \frac{1}{K}\sum_{k=1}^{K}f(G^{\prime}_{1,k},s,\tau).  $$

Substituting (20) into the summation of (22) proves the theorem.



With both Theorems 2 and 3, Algorithm 2 can be improved to Algorithm 3 which overcomes problems of large network and unknown *τ*.

In Algorithm 3, we only consider *τ* ^′^ ranging from 1 to |*A*_*τ*_| because 1) $\epsilon _{G^{\prime }_{1,k}}(s)$ ranges from 1 to |*A*_*τ*_|−1 given |*A*_*τ*_|>1 and $s\leadsto A_{\tau }\subseteq G^{\prime }_{1,k}$; 2) if *τ*>=|*A*_*τ*_|, the cascade must have terminated, thus ∀*τ*>|*A*_*τ*_|,Pr(*A*_*τ*_|*G*,*s*,*τ*)=Pr(*A*_*τ*_|*G*,*s*,|*A*_*τ*_|). The input time range [*τ*_*l*_,*τ*_*u*_] represents limited knowledge of *τ*. If the exact starting time of the cascade is known, we can use *τ*_*l*_=*τ*_*u*_=*τ*. On the contrary, if nothing at all is known about *τ*, $\tau _{l}=\min _{i}\epsilon _{G^{\prime }_{1}}(i)$ and *τ*_*u*_=|*A*_*τ*_| may be used instead.

It should be noted that for any sample $G^{\prime }_{1}$, line ?? in Algorithm 3 can be done in $O(|G^{\prime }_{1}|)$ time. First, condensation $C(G^{\prime }_{1})$ is calculated, which needs linear time. Then, since $C(G^{\prime }_{1})$ is a directed acyclic graph, there is at least one strong component in $C(G^{\prime }_{1})$ that has no predecessor. If there is exactly one such component, it is the set ; if there is more than one, $\mathcal {C} = \varnothing $. This method also applies to line ?? in Algorithm 1 and line ?? in Algorithm 2.

## Experimental results

**Fig. 2:**
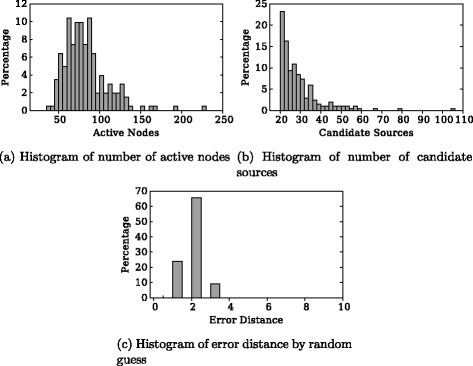
Statistics of cascades used in experiments. **a** Histogram of number of active nodes. **b** Histogram of number of candidate sources. **c** Histogram of error distance by random guess

In this section, we conduct experiments of our cascade source inference algorithm (Algorithm 3, with *K*=10^6^) on real network dataset. The network used is from WikiVote dataset ([12, 13]), which consists of all Wikipedia voting data from the inception of Wikipedia till January 2008. The dataset has 7115 nodes and 103,689 directed unweighted edges. Each node represents a Wikipedia user participating in elections, while each directed node (*i*,*j*) means user *i* voted for user *j*. We use this unweighted dataset because we cannot find a social network dataset with influence probability available despite our best effort. Since the dataset is unweighted, we use reciprocal of in-degree of the destination node as the weight of an edge. With uniformly randomly chosen source nodes, cascades are then generated on the network according to the IC model. To make the experiment challenging, we discard cascades with less than 20 candidate sources. Here, candidate source set is not active nodes set *A*_*τ*_, but set of nodes from which all active nodes are reachable in *G*_1_, i.e., {*i*∣*i*⇝*A*_*τ*_⊆*G*_1_}. We use 200 cascades in our experiments. Figure 2 a, b shows histograms of the number of active nodes and candidate sources among these cascades.

To compare our proposed algorithm with existing algorithm, we also implement the algorithm proposed by [9]. In that paper, they proposed three algorithms (“DP”, “Sort”, and “OutDegree”) to find a set of *k* sources. In our case where single source generates the cascade, their DP algorithm and Sort’ algorithm are equivalent. In the experiment below, we use this algorithm and call it “Effector” algorithm.

**Fig. 3:**
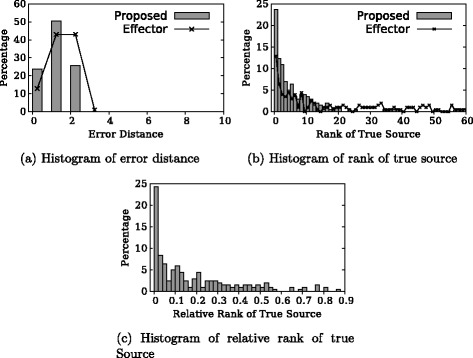
Experimental result: *τ*=|*A*_*τ*_|, *τ* known. **a** Histogram of error distance. **b** Histogram of rank of true source. **c** Histogram of relative rank of true source

First, we take snapshot at *τ*=|*A*_*τ*_|, i.e., after cascades terminate and do the experiment with exact knowledge of *τ*. Figure 3 a shows the distribution of error distances, which is defined as the distance between inferred source node and true source node assuming edges are undirected. To compare with, the error distance of random guess among *A*_*τ*_ is also shown in Fig. 1 c. It is clear that all source nodes inferred by our algorithm are within two hops around the source node, and 24 % of the inferred nodes are true sources. In comparison, the Effector algorithm has fewer results with 0 or 1 error distance. To further evaluate the algorithm, we make the algorithms output a list of candidate source nodes sorted in descending order of likelihood, rather than merely the most likely source node. This output is sometimes more useful because it answers queries like “what’s the 5 most likely source of the cascade”. Figure 3 b shows the distribution of rank of the true source node in the ordered list. In more than half of total experiments, the true source is among top 4 candidates output by our algorithm. The Effector algorithm, however, has a much heavier tail with far less results with lower ranks. In fact, there are 15 % of the results with a rank higher than 60 which is not shown in the figure. Figure 3 c shows distribution of relative ranks, i.e., rank divided by candidate set size. Only our algorithm is shown in this figure because the Effector algorithm does not calculate candidate set and their output list include many nodes not in the candidate set due to the reason explained by Fig. 1 in the ‘Introduction’ section. In more than 50 % of the experiments, our output that has relative rank of the true source is less than or equal to 0.1.

**Fig. 4:**
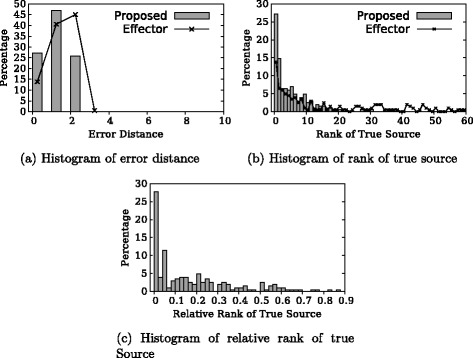
Experimental result: *τ*=8, *τ* known. **a** Histogram of error distance. **b** Histogram of rank of true source. **c** Histogram of relative rank of true source

Then, we do experiments with snapshots taken at *τ*=8, when most of the cascades are yet to terminate. The results are shown in Fig. 4. Similarly, our proposed algorithm performs better than the Effector algorithm. In 55 % of the experiments, our algorithm has true source node among top 4 candidates, and in half of experiments, we have true source node with relative rank no larger than 0.1.

**Fig. 5:**
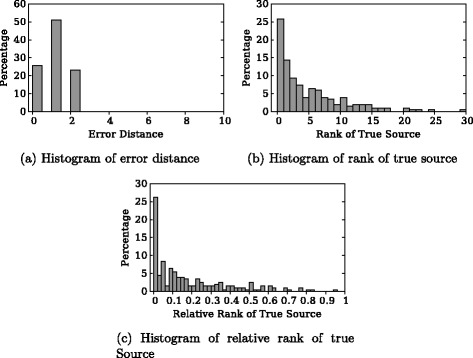
Experimental result: *τ*=8, *τ* unknown, *τ*_*l*_=0, *τ*_*u*_=16. **a** Histogram of error distance. **b** Histogram of rank of true source. **c** Histogram of relative rank of true source

To evaluate the performance of our source inference algorithm when exact cascade starting time is absent, we conduct another experiment on the snapshot taken at *τ*=8 with input time range [0,16]. As shown in Fig. 5, our algorithm effectively infers the source nodes even without exact knowledge of cascade starting time. In the experiment, 57 % of the true source nodes are among top 4 candidates, and in half of the cases, the true source ranked top 10 % in the output list.

## Conclusion

We considered cascade source inference problem in the IC model. First the #P-completeness of this problem was proven. Then, a Markov chain Monte Carlo algorithm was proposed to approximate the solution. Our algorithm was designed with two major advantages: 1) it scales with the observed cascade snapshot rather than the whole network and thus is applicable to enormous modern social networks and 2) it does not require any knowledge about the starting time of the cascade, which is a common and practical scenario in cascade source inference problem. To demonstrate the performance of our algorithm, experiments on real social network were conducted. As shown above, our algorithm performs well no matter when the cascade snapshot is taken or whether the cascade starting time is known. In all these experiments, around 25 % of the true sources are correctly identified, about half of them are among the top 4 or top 10 % of the candidates.

## Appendix 1

### Proof of Theorem 1

We will prove Theorem 1 by constructing a polynomial-time Turing reduction from s-t connectedness problem to source inference problem. S-t connectedness problem is given a directed graph $\hat G(\hat V,\hat E)$ and two nodes $s,t\in \hat V$, output the number of subgraphs of $\hat G$ in which there is a path from *s* to *t*, i.e.,$~~\text {Connectedness}(\hat G,s,t)=|\{\hat E^{\prime }\subseteq \hat E \mid | s \leadsto t \subseteq \hat E^{\prime }\}$. This problem is known to be #P-complete [11].

**Fig. 6:**
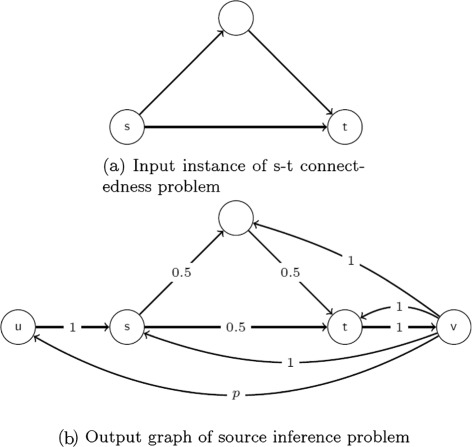
Example of Algorithm 4. **a** Input instance of s-t connectedness problem. **b** Output graph of source inference problem



A key part of the proof is Algorithm 4 which converts an instance of s-t connectedness problem to an instance of source inference problem with properties listed in Lemma 2. An simple example of this algorithm is shown in Fig. 6.

#### **Lemma2**.

Given input parameter *p* and instance $\hat G(\hat V,\hat E)$, $s,t\in \hat V$, the output instance *G*(*V*,*E*), ***w***, *A*_*τ*_, *τ* of Algorithm 4 has the following properties:

1. Pr (*A*_*t*_|*G*,*v*,*τ*)=Pr (*A*_*t*_|*G*,*t*,*τ*)=*p*;
2. $\text {Pr}\,(A_{t}|{G,i,\tau } < p, \forall i\in \hat V,i\neq t$;
3. $\text {Pr}\,(A_{t}|{G,u,\tau }) = \text {Connectedness}(\hat G,s,t) \cdot 0.5^{|\hat {E}|}$.

#### *Proof*.

In this proof, we use *i*⇝*j*⊆*G* to denote the existence of a path from *i* to *j* in graph *G*. In addition, *i*⇝*V*⊆*G* means ∀*j*∈*V*,*j*≠*i*,*i*⇝*j*⊆*G*.

According to the algorithm, output snapshot *A*_*τ*_ contains all vertices, and *τ*=|*V*| guarantees that $d_{G^{\prime }}(i,j)<\tau $ if *i*⇝*j*⊆*G* ^′^. Therefore, due to (3), the output instance has

$$\text{Pr}\,(A_{\tau}|{G,i,\tau} = \Pr \,(i \leadsto V \subseteq G^{\prime}), $$

which means considering reachability rather than distance is sufficient in the remaining part of the proof.

Now, due to line ?? in Algorithm 4, every node in $\hat V$ is reachable from *v* in every subgraph *G* ^′^ sampled via (1). And because *w*_*t*,*v*_=1 (by line ??), for any subgraph *G* ^′^,

(23) $$\begin{array}{*{20}l} &t \leadsto \hat V \subseteq G^{\prime},\\ &v \leadsto \hat V \subseteq G^{\prime},\\ &\forall i \in V, \quad i \leadsto t \subseteq G^{\prime} \Longleftrightarrow i \leadsto v \subseteq G^{\prime} \Longleftrightarrow i \leadsto \hat V \subseteq G^{\prime}. \end{array} $$

Thus property 1 is straightforward:

$$\begin{aligned} \text{Pr}({A_{t}}|{G,t,\tau}) &= \text{Pr}\,(A_{t}|{G,v,\tau})\\ &= \text{Pr}\,({v \leadsto V \subseteq G^{\prime}})\\ &= \text{Pr}\,({v \leadsto u \subseteq G^{\prime}})\\ &= p. \end{aligned} $$

On the other hand, since the new node *u* has only one incoming edge (*v*,*u*), we have $\forall i \in \hat V, i \neq t$, *i*⇝*u*⊆*G* ^′^ implies *i*⇝*t*⊆*G* ^′^. Therefore, we have the proof for property 2: for any $i\in \hat V, i\neq t$,

$$\begin{aligned} \text{Pr}(A_{t}|{G,i,\tau}) &= \text{Pr}\,({i \leadsto V \subseteq G^{\prime}})\\ &= \text{Pr}\,({i \leadsto t \subseteq G^{\prime}}) \cdot p\\ &<p, \end{aligned} $$

where the last inequality is because every incoming edge of *t* has weight 0.5 according to line ?? in Algorithm 4.

To prove property 3, we first note that *s* is the only successor of *u* and *w*_*u*,*s*_=1, with (23), we have

$$u \leadsto V \subseteq G^{\prime} \Longleftrightarrow s \leadsto t \subseteq G^{\prime}.  $$

And therefore,

(24) $$ \text{Pr}\,({A_{t}}|{G,u,\tau}) = \text{Pr}\,({u\leadsto V\subseteq G^{\prime}}) = \text{Pr}\,({s\leadsto t\subseteq G^{\prime}}).  $$

Because $\hat G \subset G$, sampling subgraphs *G* ^′^ of *G* can be viewed as sampling subsets of $\hat E$ followed by sampling subsets of $E\setminus \hat E$. Since any path from *s* to *t* consists only edges in $\hat E$, Pr (*s*⇝*t*⊆*G* ^′^) is fully determined by sampling $\hat E$, or equivalently, sampling subgraphs of $\hat G$. As a result,

(25) $$ \text{Pr}\,({s\leadsto t\subseteq G^{\prime}}) = \text{Connectedness}({\hat G,s,t}) \cdot 0.5^{|\hat {E}|},   $$

because every subset of $\hat E$ has probability $0.5^{|\hat E|}$ to be selected via (1) according to line ?? in Algorithm 4. Now property 3 follows from (24) and (25).

#### *Proof*.

First, to show source inference problem is in #P, we note that calculating Pr(*A*_*t*_|*G*,*i*,*τ*) is in #P since it is the sum of probabilities of all subgraphs of *G* with *i*⇝*V*⊆*G*. So source inference problem, i.e., finding node *i* that maximize Pr(*A*_*t*_|*G*,*i*,*τ*), is also in #P.

Since graph $\hat G$ has $2^{|\hat E|}$ subgraphs, $\text {Connectedness}{\hat G, s, t}$ must be an integer in range $[0, 2^{|\hat E|}]$. Therefore, Pr (*A*_*t*_|*G*,*u*,*τ*) of the output instance of Algorithm 4 must be in set $\{k \cdot 0.5^{|\hat E|}\mid k\in \mathbb N, k \le 2^{|{\hat E}|}\}$. A binary search algorithm, i.e., Algorithm 5, can solve s-t connectedness problem by solving source inference problem.



In Algorithm 5, there will be $|\hat E|$ iterations of while-loop. Hence, only polynomial number of queries to the oracle will be made. All other operations can be done in polynomial time. Therefore, this algorithm shows a polynomial-time Turing reduction from s-t connectedness problem to source inference problem. Since s-t connectedness problem is #P-complete and source inference problem is in #P, Theorem 1 is proven.

## Appendix 2

### Proof of Theorem 2

The proof is shown from Eqs. (26) to (33). Here, Eq. (27) follows from (15); (28) is due to the equivalence between sampling *G* ^′^⊆*G* and sampling $G^{\prime }_{k}\subseteq G_{k}, k=1,2,3$, separately; (29) results from Corollary 2 and the fact that $\text {Pr}_{\mathcal {G}_{k}}({G^{\prime }_{k}})$ depends only on $G^{\prime }_{k}$ respectively; (30) is simply due to $\sum _{G^{\prime }_{3}\subseteq G_{3}} \text {Pr}_{\mathcal {G}_{3}}({G^{\prime }_{3}}) = 1$; (31) is by Corollary 1.

To further transform the (32), we split *G*_2_ to *G*_2,*τ*_(*V*,*E*_2,*τ*_) and $G_{2,\hat \tau }(V,E_{2,\hat \tau })$, where

$$\begin{array}{*{20}l} E_{2,\tau} = \bigcup_{\substack{i\in A_{\tau}\\ d_{G^{\prime}_{1}}(s,i) = \tau}} E_{2,i},\\ E_{2,\hat\tau} = \bigcup_{\substack{i\in A_{\tau}\\ d_{G^{\prime}_{1}}(s,i) < \tau}} E_{2,i}. \end{array} $$

Then, with given subgraph $G^{\prime }_{1}\subseteq G_{1}$, sampling subgraph $G^{\prime }_{2}\subseteq G_{2}$ is essentially sampling $G^{\prime }_{2,\tau }\subseteq G_{2,\tau }$ and $G^{\prime }_{2,\hat \tau }\subseteq G_{2,\hat \tau }$, which leads to (34). Since the first summation in (34) is the sum probability of all possible subgraphs of *G*_2,*τ*_, which is 1, we have (35). Because only one specific subgraph $G^{\prime }_{2,\hat \tau } \subseteq G_{2,\hat \tau }$, namely, $G^{\prime }_{2,\hat \tau } = G_{2,\hat \tau }$, satisfies $\forall (i,j)\in G_{2,\hat \tau }$, $I((i,j)\notin G^{\prime }_{2,\hat \tau }) > 0$, we have (36). Then, substituting (36) into (31) gives (32). According to the definition of distribution $\mathcal {G}^{\prime }_{1}$, we have (33) and prove Theorem 2.

(26) $$\begin{array}{*{20}l} {}\text{Pr}\,({A_{\tau}}|{G,s,\tau}) =&\sum_{G^{\prime}\subseteq G} \text{Pr}_{\mathcal{G}}({G^{\prime}}) I(A_{\tau} = \mathcal{R}(G^{\prime},s,\tau)) \end{array} $$

(27) $$\begin{array}{*{20}l} =&\sum_{G^{\prime}\subseteq G} \prod_{k=1}^{3}\text{Pr}_{{\mathcal{G}}_{k}}({G^{\prime}_{k}}) I(A_{\tau} = \mathcal{R}(G^{\prime},s,\tau)) \end{array} $$

(28) $$\begin{array}{*{20}l} =&\sum_{G^{\prime}_{1}\subseteq G_{1}} \sum_{G^{\prime}_{2}\subseteq G_{2}} \sum_{G^{\prime}_{3}\subseteq G_{3}} \prod_{k=1}^{3}\text{Pr}_{\mathcal{G}_{k}}({G^{\prime}_{k}}) I(A_{\tau} = \mathcal{R}(G^{\prime},s,\tau)) \end{array} $$

(29) $$\begin{array}{*{20}l} =&\sum_{G^{\prime}_{1}\subseteq G_{1}} \left[\text{Pr}_{\mathcal{G}_{1}}({G^{\prime}_{1}}) \cdot\sum_{G^{\prime}_{2}\subseteq G_{2}} \left[\text{Pr}_{\mathcal{G}_{2}}({G^{\prime}_{2}}) I(A_{\tau} = \mathcal{R}(G^{\prime},s,\tau)) \cdot\sum_{G^{\prime}_{3}\subseteq G_{3}} \left[ \text{Pr}_{\mathcal{G}_{3}}({G^{\prime}_{3}}) \right] \right] \right] \end{array} $$

(30) $$\begin{array}{*{20}l} =&\sum_{G^{\prime}_{1}\subseteq G_{1}} \left[\text{Pr}_{\mathcal{G}_{1}}({G^{\prime}_{1}}) \cdot\sum_{G^{\prime}_{2}\subseteq G_{2}} \left[\text{Pr}_{\mathcal{G}_{2}}({G^{\prime}_{2}}) I(A_{\tau} = \mathcal{R}(G^{\prime},s,\tau)) \right] \right] \end{array} $$

(31) $$\begin{array}{*{20}l} =&\sum_{G^{\prime}_{1}\subseteq G_{1}} \left[\!\text{Pr}_{\mathcal{G}_{1}}({G^{\prime}_{1}}) I(A_{\tau} = \mathcal{R}(G^{\prime}_{1}, s, \tau)) \cdot\sum_{G^{\prime}_{2}\subseteq G_{2}}\! \left[\!\text{Pr}_{\mathcal{G}_{2}}({G^{\prime}_{2}})\!\! \prod_{(i,j)\!\in\! G^{\prime}_{2}}\! I(d_{G^{\prime}_{1}}\!(s,i) = \tau) \!\right] \!\right] \end{array} $$

(32) $$\begin{array}{*{20}l} =&\sum_{G^{\prime}_{1}\subseteq G_{1}} \left[\!\text{Pr}_{\mathcal{G}_{1}}({G^{\prime}_{1}}) I(A_{\tau} = \mathcal{R}(G^{\prime}_{1}, s, \tau)) \cdot\prod_{\substack{(i,j)\in G_{2}\\d_{G^{\prime}_{1}}(s,i) < \tau}} (1 - w_{i,j})\right] \end{array} $$

(33) $$\begin{array}{*{20}l} \propto& \;\mathbb{E}_{G^{\prime}_{1}\sim \mathcal{G}^{\prime}_{1}}\left[{ I(A_{\tau} = \mathcal{R}(G^{\prime}_{1}, s, \tau))\cdot \prod_{\substack{(i,j)\in G_{2}\\d_{G^{\prime}_{1}}(s,i) < \tau}}(1-w_{i,j})}\right], \end{array} $$

where (32) is due to

(34) $$\begin{array}{*{20}l} &\sum_{G^{\prime}_{2}\subseteq G_2} \left[ \text{Pr}_{\mathcal{G}_{2}}({G^{\prime}_2}) \prod_{(i,j)\in G^{\prime}_2} I(d_{G^{\prime}_1}(s,i) = \tau) \right]\\[-2pt] =&\sum_{G^{\prime}_{2}\subseteq G_2} \left[\left. \prod_{(i,j)\in G_2} w_{i,j}^{I((i,j)\in G^{\prime}_2)}(1-w_{i,j})^{I((i,j)\notin G^{\prime}_2)} \cdot \prod_{\substack{(i,j)\in G_{2}\\d_{G^{\prime}_1}(s,i) < \tau}} I((i,j)\notin G^{\prime}_2) \right] \right] \qquad\qquad\quad(\text{by}~(16)) \\[-2pt] =&\sum_{G^{\prime}_{2}\subseteq G_2} \left[\left. \prod_{\substack{(i,j)\in G_{2}\\d_{G^{\prime}_1}(s,i) = \tau}} w_{i,j}^{I((i,j)\in G^{\prime}_2)}(1-w_{i,j})^{I((i,j)\notin G^{\prime}_2)} \cdot \prod_{\substack{(i,j)\in G_{2}\\d_{G^{\prime}_1}(s,i) < \tau}} (1 - w_{i,j})I((i,j)\notin G^{\prime}_2) \right] \right]\\[-2pt] =& \sum_{G^{\prime}_{2,\tau}\subseteq G_{2,\tau}} \left[\prod_{(i,j)\in G_{2,\tau}} w_{i,j}^{I((i,j)\in G^{\prime}_{2,\tau})} (1-w_{i,j})^{I((i,j)\notin G^{\prime}_{2,\tau})}\right] \!\cdot \sum_{G^{\prime}_{2,\hat\tau}\subseteq G_{2,\hat\tau}} \left[\prod_{(i,j)\in G_{2,\hat\tau}} (\!1\! -\! w_{i,j})I((i,j)\!\notin G^{\prime}_{2,\hat\tau}) \right] \end{array} $$

(35) $$\begin{array}{*{20}l}[-5pt] =& \sum_{G^{\prime}_{2,\hat\tau}\subseteq G_{2,\hat\tau}} \left[\prod_{(i,j)\in G_{2,\hat\tau}} (1 - w_{i,j})I((i,j)\notin G^{\prime}_{2,\hat\tau}) \right] \end{array} $$

(36) $$\begin{array}{*{20}l} =& \prod_{(i,j)\in G_{2,\hat\tau}}(1 - w_{i,j}) . \end{array} $$
